# Experimental and DFT studies of gadolinium decorated graphene oxide materials for their redox properties and as a corrosion inhibition barrier layer on Mg AZ13 alloy in a 3.5% NaCl environment†

**DOI:** 10.1039/d1ra03495b

**Published:** 2021-06-22

**Authors:** Palaniappan N., Ivan S. Cole, A. Kuznetsov, K. R. Justin Thomas, Balasubramanian K., Sivakumar Manickam

**Affiliations:** School of Chemical Science, Central University of Gujarat India palaniappancecri@rediffmail.com; Advanced Manufacturing and Fabrication Research and Innovation, RMIT University Melbourne Victoria 3100 Australia ivan.cole@rmit.edu.au; Department of Chemistry, Universidad Técnica Federico Santa Maria Campus Vitacura Santiago Chile; Department of Chemistry, Organic Materials Laboratory, Indian Institute of Technology Roorkee Roorkee 247667 India; Department of Materials Engineering, Defence Institute of Advanced Technology (DU) Girinagar Pune India; Faculty of Engineering, Petroleum and Chemical Engineering, Universiti Teknologi Brunei Bandar Seri Begawan Be1410 Brunei Darussalam

## Abstract

Magnesium alloys are broadly used worldwide in various applications; however, the serious disadvantage of these alloys are subject to corrosion and in aggressive/corrosive environments. A coating containing gadolinium-based composite materials can increase the alloy protection by strong electron transfer between the host alloy and the lanthanide-containing protective layer. This investigation aims to develop a Gd nanorod functionalised graphene oxide material as a corrosion inhibition barrier on the Mg alloy surface. The obtained functional materials were characterised by various spectroscopy techniques. The corrosion inhibition and composite material stability were studied by the electrochemical methods. The electrochemical stability was shown to increase with the applied current. The hydrogen evolution constantly increased and the corrosion inhibition significantly improved. Also, the computational studies of the material were performed, and their results support the experimental findings. Overall, the resultant composite material's corrosion resistance and cyclic stability are improved, and it could be used as a sodium-ion battery cathode material due to its high reversibility.

## Abstract 2

Gadolinium electrostatically interacts with the epoxy group of graphene oxide. Non-bonding electrons of graphene oxide enhance electron transfer between the polarized metal alloy surface and product alloy surface from the corrosive medium.

## Graphical abstract

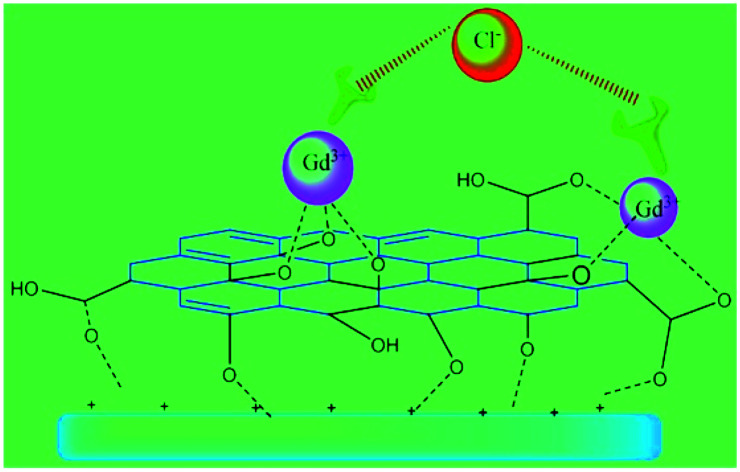

## 1. Introduction

Magnesium alloys are broadly used around the world in a variety of different applications, such as aircraft and missile component production, in the automotive industry (wheels, engine blocks, transmission cases, *etc.*), sporting equipment production, laptops, televisions, the cell phone industry, portable power tools industry, *etc.* They are considered as preferred materials when looking for weight reduction without compromising overall strength. They have also been broadly used to replace some engineering plastics due to their higher stiffness, high recycling capabilities, and lower cost of production. However, the serious disadvantage of these alloys is that they could lead to corrosion in aggressive/corrosive environments, such as saltwater, polluted air, *etc.* As a result, different methods for corrosion control and inhibition have been reported.^1^ It should be noticed that organic corrosion inhibitors are less stable in harsh environments such as strong acids/alkalis due to their poor structural stability.^2^ For instance, azole derivatives, such as benzimidazoles, possess considerable corrosion inhibition ability due to the presence of heteroatom(s).^3,5^ However, failure in their long-time stability has been observed. On the other hand, various metal complexes also possess excellent corrosion inhibition properties due to the exchange of electrons between the transition metal and inhibitor molecules, as shown recently by Zabula *et al.*^4^

Recently developed graphene oxide (GO) derivatives have been demonstrated to be excellent corrosion inhibition barriers due to their high surface area and the presence of versatile active functional groups on the surface. These functional groups assist in protecting the metal/alloy from degradation in the corrosive medium. Thus, the N- and B-doped graphenes were found to exhibit excellent corrosion resistance on the steel surface in a 3.5% NaCl environment, as observed by Ding *et al.*^6^ The corrosion inhibition of metal copper coated with silicon dioxide decorated graphene oxide was investigated in 3.5% NaCl by Sun *et al.*^7^ Without coating, the copper surface was damaged due to the diffusion of chloride ions to the surface. The synergistic effect of graphene oxide and phosphate intercalated hydrotalcite for improved anti-corrosion and self-healable protection of an epoxy coating in salt environments was shown by Chen *et al.*^8^ The first successful application of vinyl polymer/graphene-based nanocomposites in corrosion protection was reported by Yu *et al.*^9^ Further, the anti-corrosion resistance of the steel coated by aniline derivative functionalised graphene oxide in 3.5% NaCl was reported by Bagherzadeh *et al.* in 2017.^10^ They showed amine functionalised GO to have excellent corrosion resistance due to the presence of nitrogen atoms. Ramírtez Barat and Cano reported in 2019 that agarose gel coating act as a green corrosion inhibition passivation layer on the surface of statues made of iron and/or copper.^11^ Bohm and co-workers found that the graphene-coated steel improved the corrosion resistance compared with chromium coated steel. They suggested replacing chromium coating with graphene coating due to the harmful health effects of chromium.^12^ Wang *et al.*^13^ studied the corrosion inhibition in 3.5% NaCl for the mild steel coated by imidazole derived ionic liquids and epoxy functionalised graphene oxide coating. It was revealed that the steel coated with GO functionalised by imidazole derived ionic liquid had significant corrosion resistance on the steel surface. Li *et al.* conducted corrosion resistance studies in 3.5% NaCl for the steel coated by epoxy functionalised graphene oxide and graphene oxide functionalised with ionic liquids.^14^ They showed the ionic liquid decorated graphene oxide to have excellent corrosion resistance due to the presence of tails in the ionic liquid molecules, increasing the hydrophobic properties of the surface. Wang *et al.* studied corrosion inhibition in 3.5% NaCl for the steel coated by epoxy and N-decorated carbon dots.^15^ Their results revealed that the steel surface coated with N-decorated carbon dots did not form any pitting corrosion, demonstrating high corrosion resistance.

This study aims to develop gadolinium-decorated graphene oxide material as a corrosion inhibition barrier on the Mg AZ13 alloy surface in the presence of a 3.5% NaCl environment. The effectiveness of these Gd nanorod decorated GO materials is due to the electron transfer from the corrosion inhibition barrier to vacancies at the alloy surface, which improves the lifetime of the coating on the alloy surface. Also, among the Gd^3+^ species, only Gd possesses long-time redox stability. The gadolinium-decorated graphene oxide material has not been studied previously as a corrosion inhibition barrier layer on the Mg AZ13 alloy surface. It has a high potential to be used as an eco-friendly corrosion inhibition barrier material in the future.

## 2. Methods and materials

### 2.1. Material synthesis

Gadolinium nitrate (98%), NaNO_2_ (98%), KMnO_4_ (98%), H_2_O_2_ (98%), PVDF and graphite powder were purchased from Sigma Aldrich, and Mg AZ13 alloy was obtained from the local market. The graphene oxide was synthesised by Hummer's method. In brief, 3 g of graphite powder was placed in a 250 ml round bottom (RB) flask, and 40 ml of phosphoric acid and 50 ml of H_2_SO_4_ were added to the flask. The mixture was kept on ice, and 3 g of KMnO_4_ was slowly added to the mixture. While adding an oxidising agent, the temperature was increased, and the mixture was cooled down to room temperature. The mixture was then kept at 60 °C for 24 h under reflux. After 24 h, the mixture was poured into 1000 ml of deionised water, and 15 ml of H_2_O_2_ was slowly added to the mixture to stop the reaction. Then, the mixture was centrifuged at 5000 rpm. The settled black precipitate was collected and washed with ethanol, and the obtained final black product was dried at 80 °C in a vacuum oven for 24 h.

### 2.2. Characterisation

The functionalised materials were characterised to confirm the presence of non-covalent interactions with the graphene oxide matrix. The functional groups of GO and GO + Gd were studied by FTIR spectroscopy (Spectrum 65 PerkinElmer). The disorder in graphitic carbon was studied by confocal Raman spectroscopy (Wintec, laser vibration at 532 nm). The graphitic GO material crystallinity was studied by Bruker D8 Advance XRD. The functionalised carbon material microstructures were characterised by microscopy employing Carl ZEISS and EDX field emission scanning electron microscopy (FESEM) at 3.1 kV for imaging and 10 kV for EDX. Further, the Gd nanorod functionalised GO was studied by TEM FEI Tecnai F20 equipped with EDX at 200 kV. The epoxy and GO + Gd modified sheet coated alloy grain boundary were studied by the CrystTBox software to analyse the destruction of the crystal lattice of the alloy.

### 2.3. Electrochemical and corrosion inhibition studies

The electrochemical studies of the functionalised GO were carried out using three-electrode systems. The cyclic voltammetry study was done using a platinum disk electrode with a 5 mm surface as a working electrode. The active materials and PVDF with 8.2% (wt%) dissolved in NMP paste slurry form were coated on the electrode surface and dried at 80 °C for 24 h. The reference electrode was the Ag/AgCl saturated potassium chloride electrode, and the counter electrode was a platinum wire of 2 mm thickness. Aqueous KOH (1 M) was used as an electrolyte. Corrosion studies were performed in 3.5% NaCl. The working electrode was prepared from Mg AZ13 alloy, which was cut into 1 cm × 1 cm squares and polished with different grades of emery paper from 600 to 2000 to achieve a grade of the polished mirror and then washed with deionised water. The corrosion inhibition studies were performed after five days of immersion in the corrosion medium, and the experiments were repeated three times to check the reproducibility. The corrosion inhibition efficiency was evaluated using eqn (1) and (2). The redox and hydrogen evolution studies were performed in the above-described electrochemical system. The working electrode was a platinum disc electrode, and the scan rate employed was 10 mV s^−1^. The voltage was applied from the open circuit potential.1
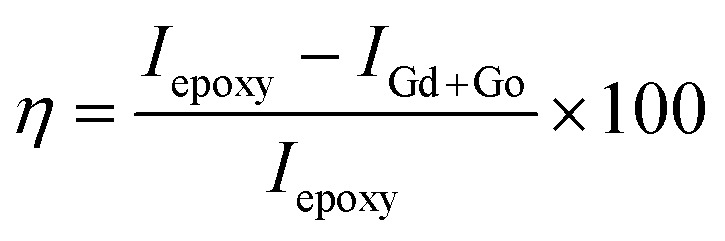
2
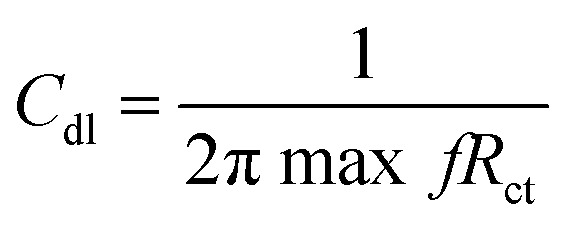
where *I*_epoxy_ is for the epoxy coated Mg alloy, *I*_Gd+GO_ is for the GO–Gd coated alloy, *R*_ct_ is the charge transfer resistance.

### 2.4. Computational methods

The C_44_H_16_O_7_GdCl_3_ graphene oxide model was used, containing two –CO_2_H groups, two –OH groups, one =O group, and the GdCl_3_ group is attached to the graphene oxide surface through the O-linkage. The protonated model with H^+^ bound to the O-linkage between Gd and graphene was studied to elucidate the corrosive acidic medium effects on the Gd-modified GO. Computational investigation of this model structure was performed using the Gaussian 09 package, revision B.01.^16^ All calculations were carried out using the hybrid DFT functional B3LYP^17^ and SDD basis set, which includes pseudopotential for Gd.^18^ We optimised the structures, calculated frequencies, and performed MOs and NBO analysis^19,20^ of our models by taking into account the implicit effects from water (dielectric constant, *ε* = 78.3553). With the implicit solvent effects, all calculations were done using the self-consistent reaction field IEF-PCM method^21^ (UFF default model used in the Gaussian 09 package, with the electrostatic scaling factor *α* set to 1.0).^22^ For the global reactivity analysis, the global electrophilicity *χ*, global hardness *η*, global softness *σ*, and nucleophilicity *ω* values were calculated based on the eqn (3)–(6) (where *I* is the ionisation potential and *A* is the electron affinity).^23^3
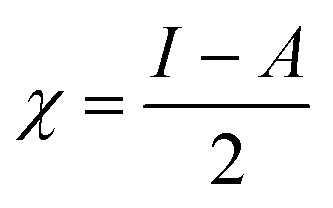
4
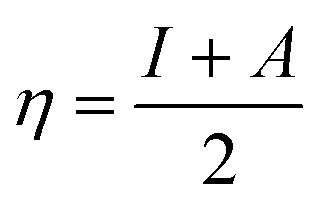
5
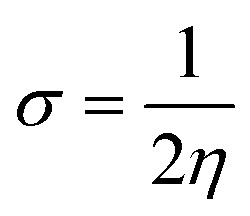
6
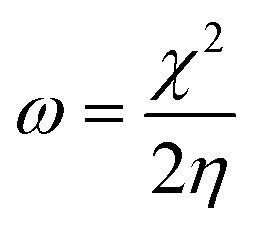


OpenGL version of Molden 5.8.2 software was utilised for the structures and FMOs visualization.^24^

Also, to check the suggested scheme of the Gd-coordination to the GO sheet, we performed the calculations of the larger model, C_56_H_18_O_10_GdCl_3_, where the GdCl_3_ group is bound to four =O groups on the GO model, using the B3LYP functional and the Stevens/Basch/Krauss ECP (effective core potential) minimal basis set CEP-4G^25,26^ (we had to employ this basis set due to the computational demands of these calculations), both in the gas phase and with the implicit effects of water included. In these studies, Gaussian 16, version B.01, was used.^27^

## 3. Results and discussion

### 3.1. Spectroscopy studies of GO and GO + Gd composite materials

**Fig. 1:**
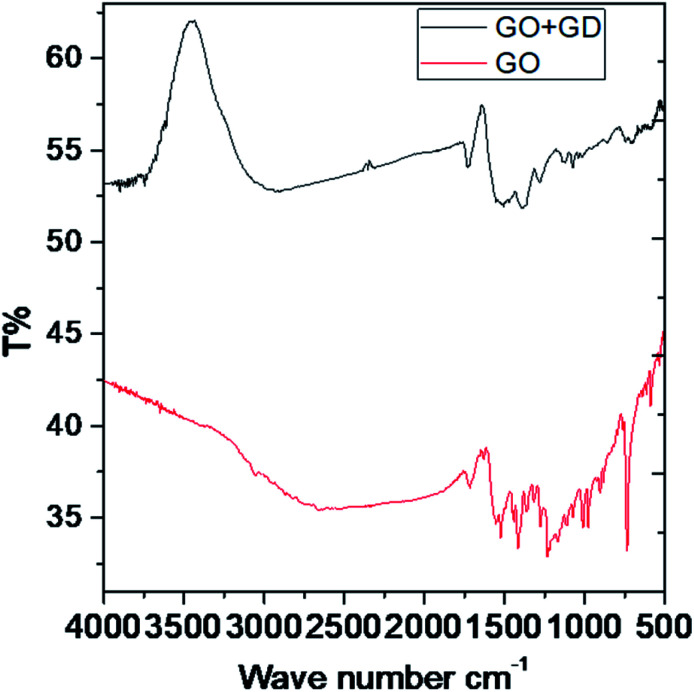
FTIR spectra of the GO and GO + Gd composite.

In the IR spectra, the stretching frequency appearing at 3500 cm^−1^ is due to the vibrations of the edge hydroxyl group of GO, as shown in Fig. 1.

**Fig. 2:**
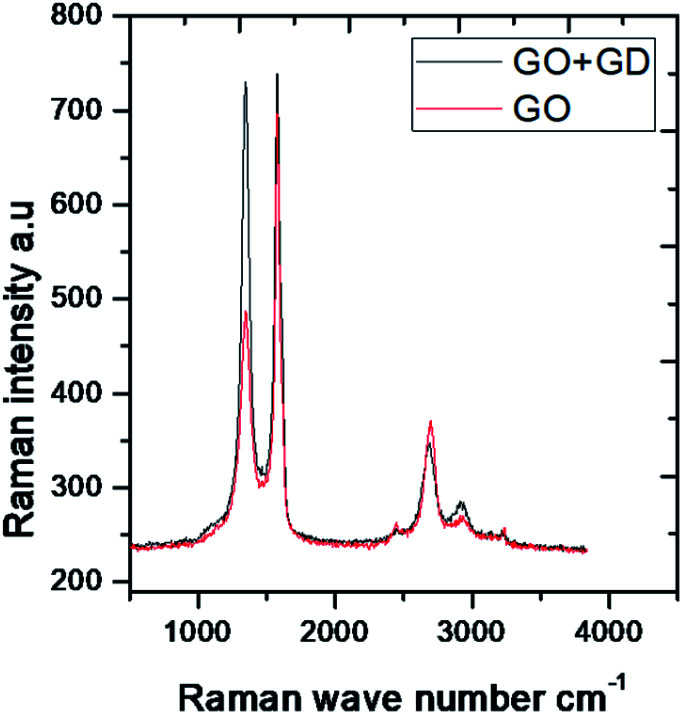
Raman spectra of GO and GO + Gd composite.

The aromatic C=C bond vibration appears at 2500 cm^−1^ (stretching frequency). The peaks around 1730 and 1520 cm^−1^ are associated with carbonyl groups of graphene oxide. The stretching frequency of Gd functionalised graphene oxide observed around 2400 cm^−1^ is due to the bond vibration of the gadolinium complex.^28,29^ The carbonyl peaks at 1700 cm^−1^ and 1600 cm^−1^ disappear due to the coordination of gadolinium with carbonyl groups. As shown in Fig. 2, GO peak around 1300 cm^−1^ is associated with D peak, and peak appearing at 1600 cm^−1^ is associated with G peak. However, new peaks could be found around 2600, 2800 and 3500 cm^−1^ due to the 2D carbon material.^30^ The gadolinium decorated graphene oxide showed a higher percentage of D and G peaks due to surface modification. However, the *I*(D)/*I*(G) ratio was higher than that for GO.

### 3.2. Microstructure studies

**Fig. 3:**
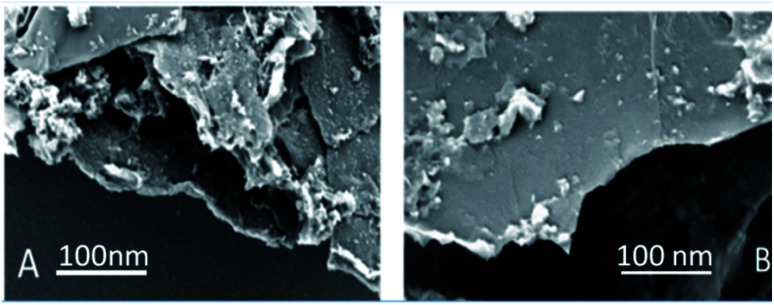
(A) GO and (B) GO + Gd decorated FESEM microstructures.

Graphene oxide, as seen in Fig. 3A, exhibits several layers exfoliated from a multilayer graphite matrix. The Gd functionalised graphene oxide possesses several layers with Gd nanorods on the graphene oxide matrix. Fig. 3B shows the thickness of the GO sheet layer around 100 nm after subjecting to continuous sonication for 3 h.

**Fig. 4:**
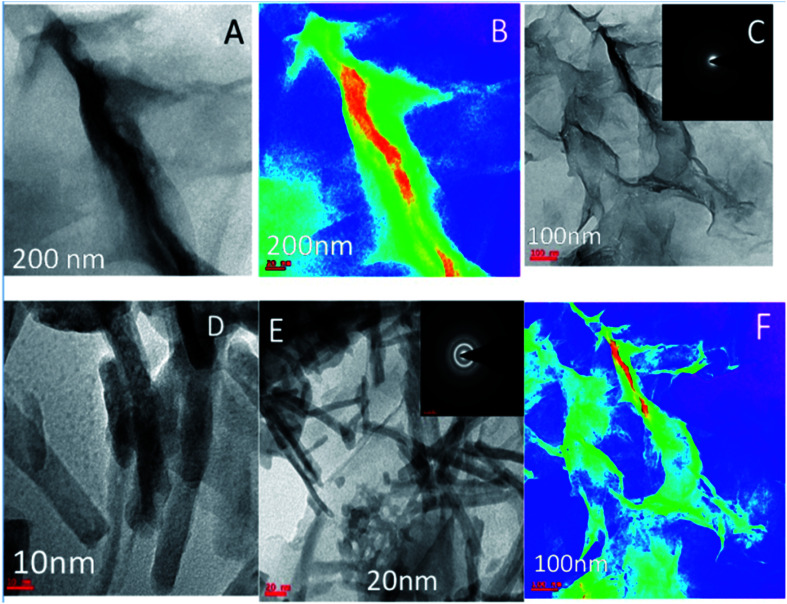
(A–C) TEM microstructure and polycrystalline structure of GO and (D–F) microstructure of Gd + GO decorated graphene oxide.

TEM image of the single graphene oxide sheet in Fig. 4A shows continuous grain boundaries. Fig. 4B illustrates the GO formation of a continuous sheet due to the action of the mild oxidising agent, and Fig. 4C reveals that the GO sheet is defectless and polycrystalline. Fig. 4D shows that Gd nanorods possess an open structure. Fig. 4E reveals that Gd nanorods are located between the GO sheets. The diffraction pattern also showed the presence of polycrystalline material.^31^ Further, Fig. 4F indicates that the GO sheets are twisted. We further confirmed the Gd functionalised GO structure where Gd nanorods were incorporated into graphene oxide, and the length of Gd nanorods is around 10 nm.

### 3.3. Mechanism of Gd coordination on the GO sheet

**Fig. 5:**
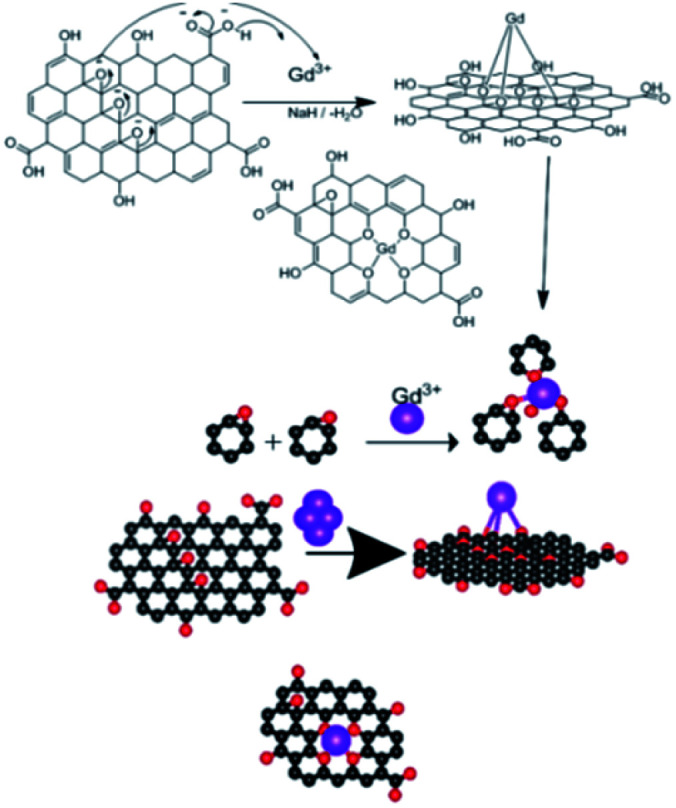
Proposed scheme of Gd^3+^ coordination with the GO sheet.

The epoxy group of graphene oxide is the key for chelation with Gd^3+^, where the non-bonding electron of epoxy oxygen is shared with Gd^3+^ cations,^32,33^ as shown in the proposed scheme (Fig. 5). This is supported by DFT calculations on the larger GO model (see below).

Our goal is to replace the traditional organic inhibitor molecules making epoxy coating a corrosion-resisting layer on the metal alloy. The epoxy groups of graphene oxide act as the self-healing agent on the alloy surface to fulfil this.

### 3.4. Electrochemical stability studies

**Fig. 6:**
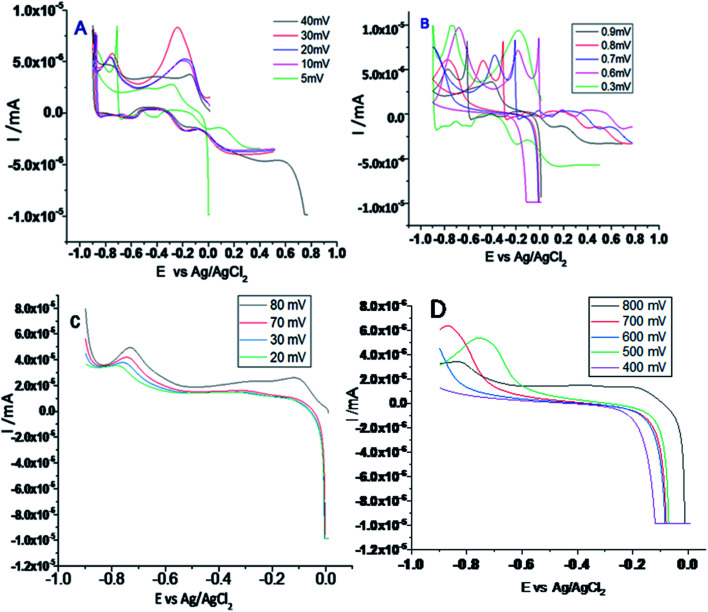
(A and B) Cyclic voltammetry of gadolinium decorated graphene oxide and (C and D) LSV studies of gadolinium doped graphene oxide.

Fig. 6A–D shows the results of electrochemical stability studies of Gd functionalised graphene oxide, which were conducted three times. As shown in Fig. 6A, electrochemical stability was studied in the voltage range from 5 to 40 mV to reveal the composite material redox behaviour. The redox behaviour was improved due to the presence of non-bonding electrons in the GO matrix, which may lead to potential applications of this material as sodium-ion battery (SIB) electrode materials.

On the other hand, as shown in Fig. 6B, with the applied voltage from 300 to 200 mV, the electrochemical redox stability, adsorption, and desorption increased due to the interactions between the graphene oxide layer and Gd^3+^ cations.^34,35^ Moreover, the exchange of electrons between gadolinium and the carbonyl and epoxy groups of GO and continuous electron transfer between GO matrix and gadolinium nanorods and the high reversibility of this process may render this material as a battery cathode material. Further, LSV studies were performed to confirm hydrogen evolution, as shown in Fig. 6C. From 20 to 80 mV, the current densities were increased. Fig. 6D shows that with an increase in the applied voltage from 400 to 800 mV, hydrogen evolution increased due to the strong interactions of Gd^3+^ ions with the graphene oxide matrix.

### 3.5. Electrode interface and hydrogen evolution studies

As shown in Fig. S1a,† the Motto–Schottky curve indicates that gadolinium species adsorb on the electrode/electrolyte interface. Therefore, with an increase in the applied frequency from 100 to 400 MHz, hydrogen evolution increased due to the intercalation of active gadolinium species over the graphene oxide matrix. Furthermore, the composite material is structurally stable, and π-electrons of graphene oxide constantly exchange with the electrons of Gd nanorods.

It has been shown that with an increase in the applied frequency, the hydrogen evolution increased due to the delocalisation of π-electrons. AC voltammetry studies of Gd decorated GO (Fig. S1b†) showed higher mass transfer between GO matrix and Gd nanorods. The applied voltage of 20 mV increases the phase due to the increased diffusion of Gd^3+^ cations to the graphene oxide interlayer. AC voltammetry phase shift is related to the diffusion of host ions to the graphene oxide matrix.^36^ The out-phase voltage shift indicates an increase in the intercalation of Gd^3+^ cations in the graphene oxide matrix. Chronoamperopotentiometry studies, as shown in Fig. S1c,† reveal the long-time cyclic stability of the studied composite material. The applied potential was changed from 10 to 800 mV at 20 cycles. Stable redox behaviour was observed at 80 mV, indicating the adsorption/desorption of gadolinium host ions on the platinum disk electrode surface. Fig. S1d† exhibits the chronocoulometry study results for the adsorption and desorption of Gd^3+^ on the working electrode surface (forward and reverse reaction). The applied voltage was changed from 10 to 900 mV s^−1^ to find the charge–discharge stability in a 1 M aqueous NaOH electrolyte at 20 cycles. No structural loss occurs due to the continued stability of the graphene oxide sheet and intercalation of Gd^3+^. The Gd^3+^ cations strongly coordinate with the edge carbonyl groups, providing high stability for the cyclic behaviour. The carbonyl and carboxyl groups of graphene oxide have been shown to enhance stability. The non-bonding electrons of graphene oxide could improve recycling behaviour during the reverse reaction.

### 3.6. Corrosion studies

**Fig. 7:**
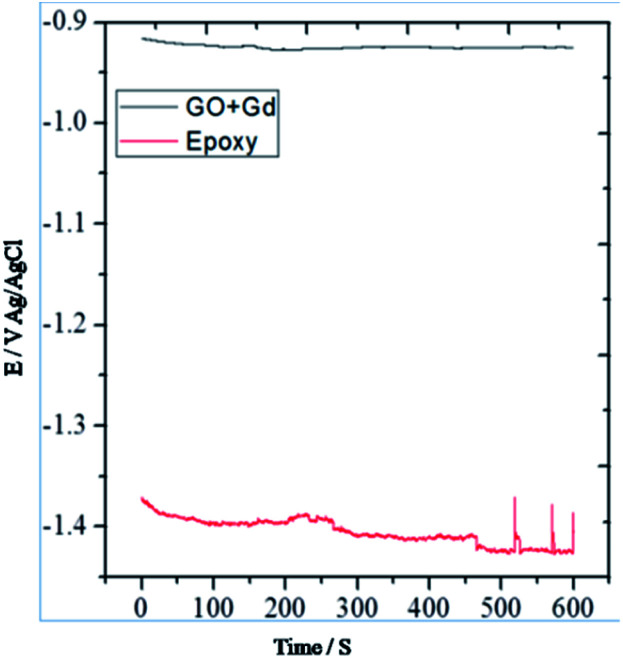
OCP studies of epoxy and Gd decorated GO coated Mg alloys.

From Fig. 7 it could be noted that the epoxy-coated Mg alloy shows more negative potential compared to the Gd + GO coated Mg alloy due to the occurrence of localised corrosion.

**Fig. 8:**
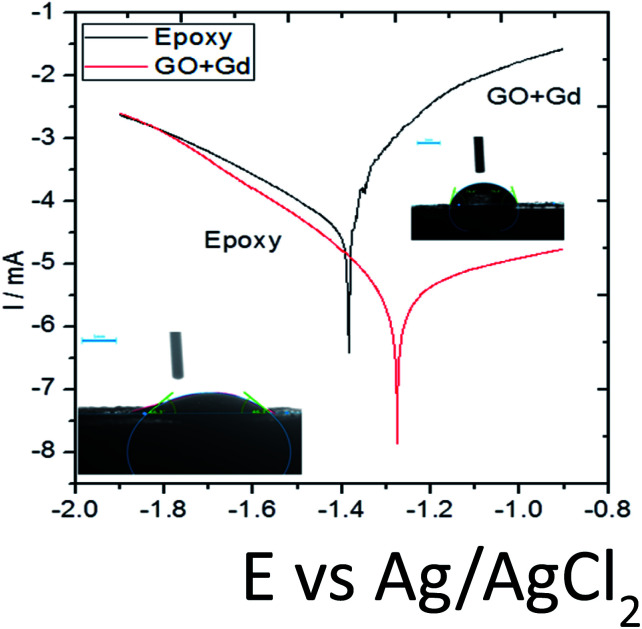
Potentiodynamic studies for the epoxy and Gd doped graphene oxide coated Mg alloy immersed in 3.5% NaCl medium.

On the other hand, Gd + GO coated Mg alloy shows more positive potential attributed to the Gd^3+^ functionalised GO sheet forming an impermeable layer and thus preventing corrosive ions from reaching the alloy surface.^37^ As can be seen in Fig. 8, potentiodynamic polarisation studies revealed that the epoxy-coated Mg alloy has a higher *I*_corr_ relative to the Gd + GO coated Mg alloy.

Further, the epoxy coated alloy surface lower wettability (46.3°) is due to the penetration of aggressive ions to the epoxy coating. The corrosion current of epoxy coated Mg alloy *I*_corr_ was significantly higher than for the Gd + GO coated Mg alloy. For the Gd + GO coated alloy, the corrosion current was lower as the gadolinium decorated graphene oxide was strongly physisorbed on the alloy surface. The *E*_corr_ was increased for the Gd + GO coated Mg alloy compared to the epoxy coated alloy due to gadolinium functionalised graphene oxide where the edge functional groups of graphene oxide such as carboxyl and hydroxyl anions coordinate with Gd^3+^ cations.

**Fig. 9:**
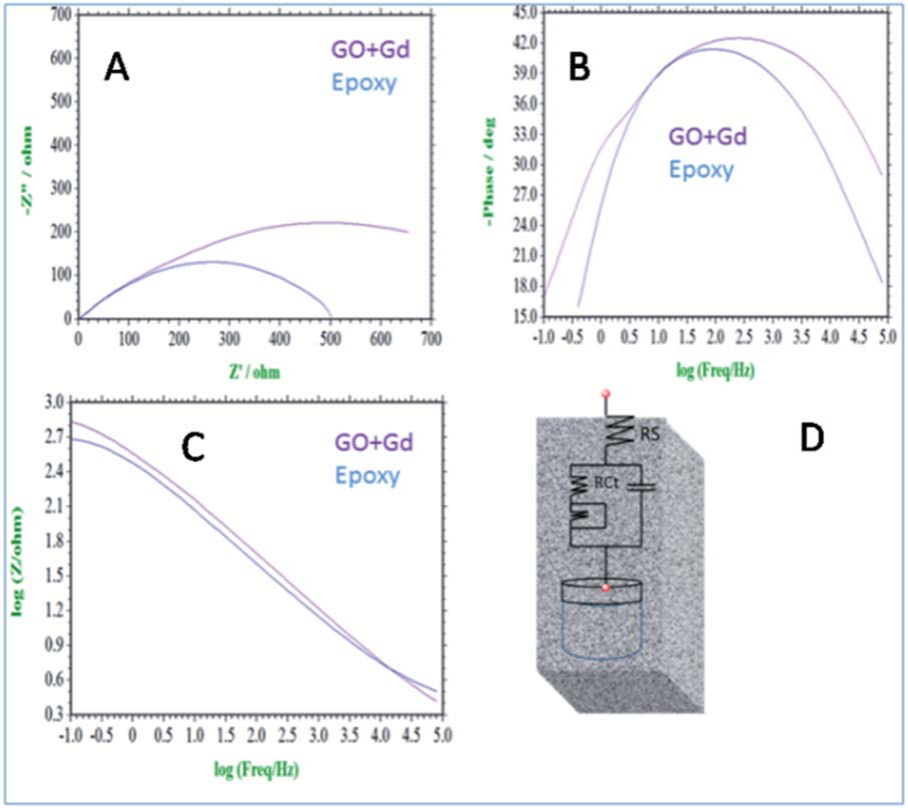
(A) Nyquist spectrum (B) phase impedance (C) Bode impedance and (D) equivalent circuit impedance spectroscopy of epoxy and Gd decorated graphene oxide coated Mg alloy immersed in 3.5% NaCl.

Further, the surface wettability for the GO + Gd coated Mg alloy surface was found to be around 74.3°, noticeably higher as compared with the epoxy coated surface. The linear polarisation of the GO + Gd coated Mg alloy calculated using CHI920D software was also significantly higher than for the epoxy coated Mg alloy (1408 A m^−2^ against 382 A m^−2^) due to the high surface wettability of the GO + Gd coated surface.^38–41^ The results of the impedance spectroscopy studies of the epoxy coated and Gd decorated GO coated alloys are shown in Fig. 9. The epoxy coated alloy exhibits highly depressed semicircles in the Nyquist spectrum, suggesting failure (Fig. 9A).^42^ As shown in Fig. 9D, for the impedance values fitted by the Randle circuit, the solution resistance *R*_S_ decreased due to the attack of corrosive ions on the alloy surface.

Also, the charge transfer resistance *R*_ct_ was decreased by aggressive ion reacting with the epoxy coating. The double-layer capacitance values were increased due to corrosion occurring on the alloy surface, and the impedance frequency and impedance phase were decreased, as shown in Fig. 9B. However, the Gd decorated GO coated alloy showed the solution resistance to increase and charge transfer resistance to increase, thus showing the composite material having excellent physisorption on the alloy surface,^43–45^ as shown in Fig. 9C. The impedance frequency plot showed a high-frequency angle due to the presence of Ga^3+^ ions on the graphene oxide. The corrosion inhibition values are presented in Table S1.† The impedance phase spectrum also showed a high phase angle due to the presence of composite materials. The charge transfer resistance values were increased by Gd^3+^ nanorod electrostatic interactions with the graphene oxide matrix, and Gd^3+^ ions decorated graphene oxide resisted corrosive ions flow.

### 3.7. Microstructure studies of alloy

**Fig. 10:**
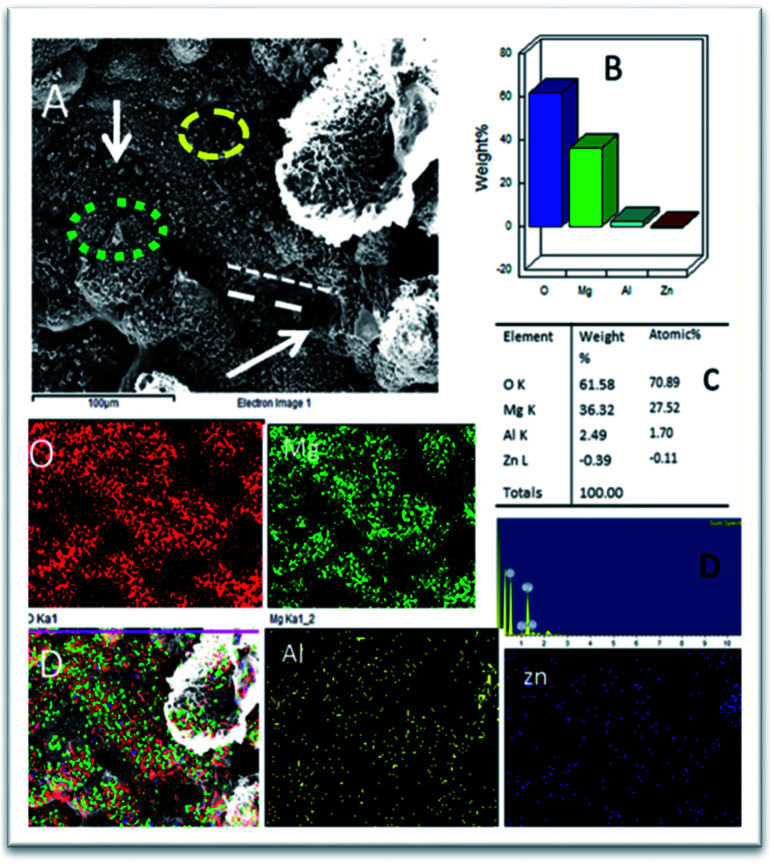
(A) Epoxy coated Mg alloy corrosion passivation studies after 5 days immersion in 3.5% NaCl solution and (B–D) weight percentage loss of Mg alloy.

As shown in Fig. 10, the epoxy-coated Mg alloy has grain boundaries severely affected by the attack of corrosive chloride ions (the affected areas are marked). The arrows show that the alloy surface exhibits hill type pitting due to the feeble epoxy coating. In Fig. 10A, the circles indicate localised corrosion attack and double lines indicate a failure in the passivation layer and enhancement in the corrosion process. Also, Fig. 10B–D indicates the percentage composition affected by the corrosive ion chlorides. The oxygen concentration is high as the alloy undergoes corrosion.^46^ The elemental percentages of Al and Zn were decreased due to the attack of corrosive chloride ions on the alloy surface.

**Fig. 11:**
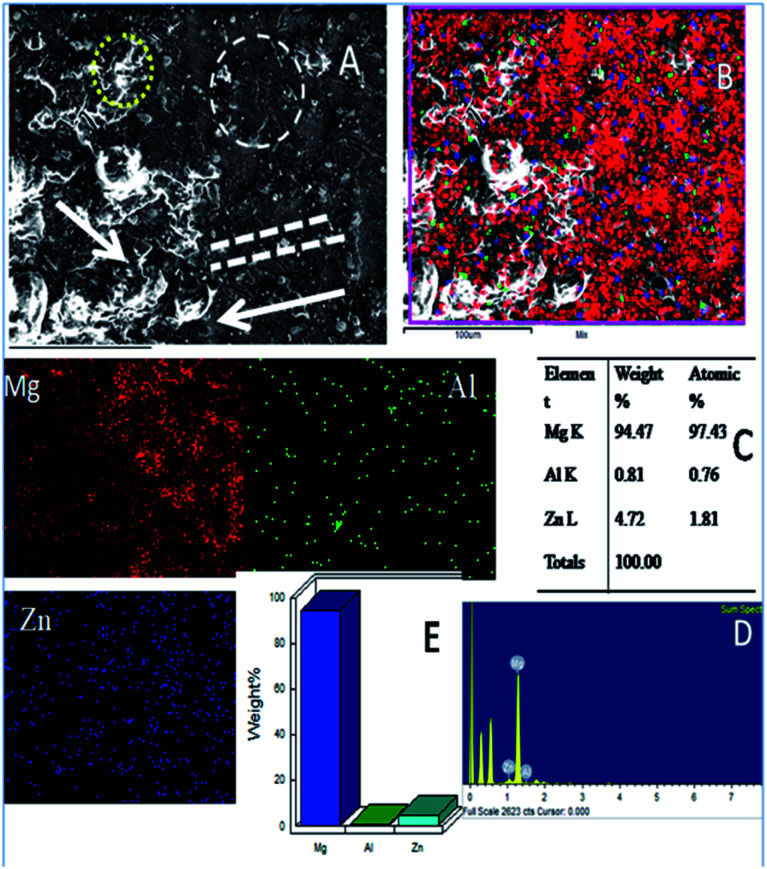
(A) Gd decorated graphene oxide coated Mg alloy after 5 days immersion in 3.5% NaCl medium (B) mixture of Mg alloy composition (C) atomic weight percentage of alloy composition and (D and E) pictorial graph representing the weight percentage loss.

Further, the microstructure of the Gd doped GO coated alloy is shown in Fig. 11. The alloy surface does not seem to have any pitting corrosion or localised corrosion due to the Gd doped GO passivation layer on the alloy. The corrosion initiation is retarded by the Gd doped graphene oxide.^47^ The coated layer displays strong passivation on the alloy surface. The carbonyl groups of the GO support electron transfer between the GO interlayer and Gd^3+^ cations. Further, circles in Fig. 11A suggest arresting localised corrosion processes as Gd^3+^ composite materials donate an electron to the active metal surface. Also, the dotted lines reveal that corrosive ions cannot penetrate the coating due to strong physisorption on the metal surface. In Fig. S2a,† for the epoxy coated Mg alloy, (111), (001), and (101) crystal lattices are shown. The polycrystalline (001) phase of the alloy is damaged, revealing the maximum area compared with other crystalline lattices (111 and 101) as it covers the phase of Mg alloy.

The grain boundary orientation could be confirmed from Fig. S2b.† Fig. S2c† suggests changes in the crystalline phase because of the attack of corrosive chloride ions. When the plane of 101 phases is damaged, the corrosive chloride ions degrade the β phase of Mg alloy.^44^ In the Gd decorated graphene oxide coated Mg alloy, and the alloy crystalline structure does not change as it is protected from the modified graphene oxide layer (Fig. S2d–f†). Fig. S2d† reveals the uniform distribution of colour mapping from three lattice planes since the grain boundary does not change. Fig. S2e† indicates that grain boundary orientation is regularly formed. Fig. S2f† concludes that lattice parameters are uniformly arranged in a line.

### 3.8. AFM studies

The topography of epoxy and Gd^3+^ doped GO coated Mg alloy immersed in 3.5% NaCl for five days is shown in Fig. S3.† The topography of the epoxy-coated Mg alloy is significantly affected by the diffusion of corrosive ions. Fig. S3a† shows local degradation caused by corrosive ion reaction with the epoxy coating, indicating that epoxy coating cannot protect the alloy for a long time in a corrosive medium and circles demonstrate the localised attack on the alloy surface, and the arrow indicates the formation of pinhole by the attack of corrosive ion.^45–48^ Fig. S3b† shows the depth of local degradation on the Mg alloy, and dotted line circles represent the attack of corrosive ion on the surface.^49–52^ Fig. S3c† indicates the appearance of a pinhole on the alloy surface due to coating failure. The maximum pitting size is around 1.0 to 1.8 Å due to the attack of chloride ions on the epoxy coating. Further, the maximum area of attack could be noted around 4.7 Å, and such an attack was widespread across the surface.^48^ Also, Mg alloy surface roughness showed hill type damages due to the initiation of the corrosion process. As shown in Fig. S4,† Gd decorated graphene oxide coated Mg alloy has a smooth surface compared with the epoxy coated Mg alloy. As shown in Fig. S4a,† the propagation of a local attack was controlled by the Gd decorated graphene oxide.

Fig. S4b† suggests that the initiation of the local attack on Gd decorated graphene oxide Mg alloys was suppressed by the Gd decorated graphene oxide, which changes the electron transfer between the coating substrate, and green colours indicate the progress of retarded corrosion on the metal alloy. From Fig. S4c,† it can be suggested that the initiation of a local attack was retarded by Gd decorated graphene oxide and also that the initiation of pitting was controlled by the Gd modified graphene oxide due to the excellent electrochemical stability of the modified graphene oxide sheet in the corrosive medium. Further, the results imply that the size of the local attack area is less than for the epoxy coated Mg alloy.

### 3.9. Computational studies

**Fig. 12:**
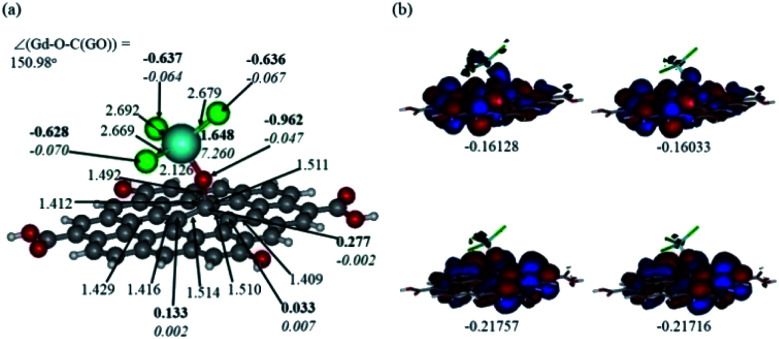
Optimised structure of the Gd-decorated GO model calculated with the implicit effects from water (a) and its α- (left column) and β- (right column) FMOs (b) (HOMOs at the bottom and LUMO at the top), with MOs energies in au. Colour coding: grey for C, light-blue for Gd, green for Cl, red for O, and white-grey for H. Distances are given in Å and angles in degrees, NBO charges are shown in bold and spin densities are shown in italics.

**Fig. 13:**
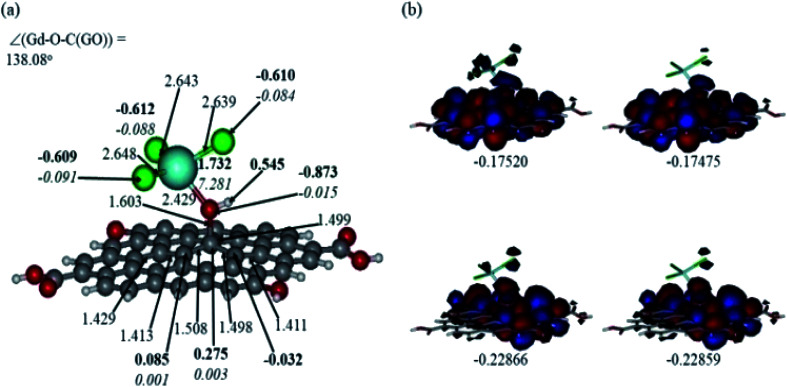
Optimised structure of the protonated Gd-decorated GO model calculated with the implicit effects from water (a) and its α- (left column) and β- (right column) FMOs (b) (HOMOs at the bottom and LUMO at the top), with MOs energies given in au. Colour coding: grey for C, light-blue for Gd, green for Cl, red for O, and white-grey for H. Distances are given in Å and angles in degrees, NBO charges are shown in bold and spin densities are shown in italics.

In Fig. 12 and 13, the structures optimised with the implicit water effects along with the frontier molecular orbitals (FMOs) of the Gd-decorated GO model with the central location of the O–GdCl_3_ moiety are shown, for the neutral and protonated models, respectively. The calculated NBO charges and spin densities (shown in bold and italics, respectively) are also given in these figures. The octet structures were calculated to be the lowest in energy in both cases, and the structures with the central location of the O–GdCl_3_ moiety were found to be lower in energy than the structures with the edge location of this group by 4.14 and 3.07 kcal mol^−1^ for the neutral and protonated models, respectively.

As can be seen in Fig. 12a, the O–Gd bond distance is quite long, 2.126 Å and the O(Gd)–C(GO) bond distance is relatively shorter, *i.e.* 1.492 Å. The monolayer of the graphene oxide model near the O-linkage bound to the GdCl_3_ moiety becomes somewhat distorted upon optimisation. Interestingly, the Gd–Cl bond distances were calculated to be unequal, varying from 2.669 to 2.692 Å. The NBO analysis shows that the Gd-centre carries a significant positive charge, *ca.* 1.65*e*, along with the significant α-spin density of 7.26*e*, accounting for most of the unpaired spin in the ^8^A model. Significant negative charges, −0.63 to −0.64*e*, along with the small amounts of β-spin density, *ca.* −0.07*e*, were calculated on the Cl-centres. The O-centre connecting Gd to the GO surface was calculated to have a noticeable negative charge, *ca.* −0.96*e*, along with a small amount of β-spin density, *ca.* −0.05*e*. The accumulation of positive charge was calculated to occur on the C-centre connected to this oxygen, *ca.* 0.27*e*, and on some of the adjacent C-centres as well. However, those carbons bear negligible amounts of spin density (Fig. 12a).

Comparison of the protonated and neutral systems (Fig. 13a and 12a, respectively) shows that upon protonation of the O-linkage connecting the GdCl_3_ group to GO, both Gd–O and O–C (GO) bond distances become elongated noticeably by *ca.* 0.30 and 0.11 Å, respectively. On the contrary, the Gd–Cl bond distances become shortened by *ca.* 0.04–0.05 Å. Also, the Gd–O–C(GO) angle upon protonation decreases significantly by *ca.* 13°. The C–C bond distances around the C-centre to which the –OGdCl_3_ moiety is connected to become somewhat shorter upon protonation. It is important to note that the relatively long Gd–O bond distances in both models may increase the accessibility of the –OGdCl_3_ moiety for the reactions with different (corrosive) agents in the ambient medium, thus preventing them from approaching the metal surface coated with the Gd decorated GO layer. Furthermore, upon protonation, the positive charge on the Gd-centre increases by *ca.* 0.08*e*, whereas the spin density increases by *ca.* 0.02*e*. The negative charge on the protonated O-linkage becomes smaller by *ca.* 0.09*e*, and β-spin density on this linkage decreases by *ca.* 0.03*e* (the proton connected to this O-linkage carries a significant positive charge, *ca.* 0.55*e*). The negative charges on the Cl-centres in the protonated system decrease by *ca.* 0.02–0.03*e*, whereas the β-spin density increases by *ca.* 0.02*e*. Interestingly, the positive charge on the C-centre to which the O-linkage is connected remains essentially the same, whereas charges on the adjacent C-centres decrease quite noticeably. Again, these changes in the charge might affect the reactivity of the –OGdCl_3_ moiety. It is also important to emphasise that high positive charges and high amount of the spin density on the Gd-centre in both models, along with their structural accessibility, would make the Gd-centre quite reactive towards various (corrosive) agents in the solution, including coordination of chloride ions.

Fig. 12b and 13b present α- and β-HOMOs and LUMOs of the neutral and protonated models, respectively. The analysis of the molecular orbitals shows the following. (i) In both models, both the GO layer and the O-centre connecting Gd to the GO surface contribute to the HOMOs. The GO layer and the O-linkage also dominate the LUMOs. (ii) The contributions from the GdCl_3_ group to the frontier MOs are not very significant for both models. (iii) The HOMO–LUMO gaps for both the neutral and protonated systems were calculated to be quite small, *ca.* 0.053–0.057 au (Table 1) or *ca.* 1.44–1.55 eV, which signifies that the Gd-decorated GO material should be quite reactive in the solution media. The HOMO–LUMO gaps slightly decrease upon protonation. (iv) The computed global hardness (*η*) values for the neutral and protonated models are quite small, *ca.* 0.03 au and the global softness *σ* values are quite significant, 35.2–37.4 au (Table 1). This suggests that the system would be quite highly reactive towards oxidising agents in the solution, which would attack the surface. Relatively small global electronegativity (*χ*) values, *ca.* 0.19–0.20 au, and higher, but still relatively low, global electrophilicity (*ω*) values, *ca.* 0.63–0.76 au, suggest that this system would have high potential reactivity with the oxidising electrophilic agents.

**Table 1:** Global reactivity parameters computed for the Gd–GO system (with the implicit effects from water), for the neutral and protonated models (au): HOMO–LUMO gap Δ*E*, ionisation potential *I*, electron affinity *A*, global electrophilicity *χ*, global hardness *η*, global softness *σ*, and global nucleophilicity *ω*. A = α-HOMO & LUMO, B = α-HOMO & LUMO

	HOMO	LUMO	Δ*E*	*I*	*A*	*χ*	*η*	*σ*	*ω*
**Neutral**									
A	−0.21757	−0.16128	0.05629	0.21757	0.16128	0.189425	0.02815	35.524	0.63733
B	−0.21716	−0.16033	0.05683	0.21716	0.16033	0.188745	0.02842	35.1865	0.62675

**Protonated**									
A	−0.22866	−0.17520	0.05346	0.22866	0.17520	0.20193	0.02673	37.4111	0.76273
B	−0.22859	−0.17475	0.05384	0.22859	0.17475	0.20167	0.02692	37.1471	0.7554

Thus, the following computational findings support experimental data, showing the noticeable suitability of the Gd-doped GO materials for being used as a very good corrosion inhibition material. (i) Structural accessibility of Gd-centres for interactions with various agents present in the solution phase, thus preventing them from approaching the surface. (ii) Significant positive charges and high amounts of the spin density on the Gd-centres, along with their structural accessibility, would make them quite reactive towards various (corrosive) agents present in the solution, including coordination of chloride ions. (iii) Small global hardness values and significant global softness values, along with relatively small global electronegativity and global electrophilicity values, suggest that the system is highly reactive towards oxidising electrophilic agents in the solution which would attack the surface.

**Fig. 14:**
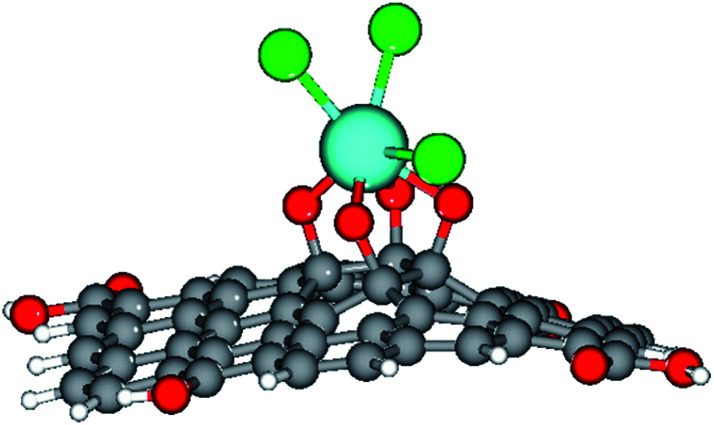
The optimised structure of the Gd-decorated GO model with 54 carbons. Colour coding: grey for C, light-blue for Gd, green for Cl, red for O, and white-grey for H.

Furthermore, our computational study supported the proposed scheme of the Gd coordination to the GO surface (Fig. 5), as shown in Fig. 14.

## 4. Conclusions

In this study, we have reported the synthesis and characterisation of the Gd functionalised graphene oxide, along with the results of the thorough investigation of its properties of hydrogen evolution, electrochemical stability, and corrosion inhibition efficiency. Various spectroscopic and electrochemical techniques, along with DFT calculations, were employed. The spectroscopy studies suggest chelation of Gd^3+^ cations with the epoxy groups of the GO matrix. Raman spectrum reveals uniform disorder of GO matrix. Also, the microscopy studies indicate the formation of single-layer graphene oxide. The corrosion inhibition efficiency of the Gd + GO coated Mg alloy was found to be 59%. The electrochemical stability study showed redox stability up to 800 mV. The theoretical studies on the composite material showed the high accessibility and reactivity of its Gd-centres towards various oxidising agents, which can be present in the solution phase, thus, confirming that the Gd-decorated GO should be quite an effective corrosion inhibition material. Hence, it can be concluded that the Gd decorated graphene oxide can be used as a novel effective material for protecting the alloys against corrosion and could also be used as a battery material.

## Conflicts of interest

There are no conflicts to declare.

## Supplementary Material

RA-011-D1RA03495B-s001
