# Supplementary material for: Experimental and DFT studies of gadolinium decorated graphene oxide materials for their redox properties and as a corrosion inhibition barrier layer on Mg AZ13 alloy in a 3.5% NaCl environment
Source: RSC Adv. 2021 Jun 22;11(36):22095–105. doi: 10.1039/d1ra03495b (PMC9034221; doi:10.1039/d1ra03495b)
Supplement: RA-011-D1RA03495B-s001 [file RA-011-D1RA03495B-s001.pdf]

Electronic Supplementary Material (ESI) for RSC Advances.
This journal is © The Royal Society of Chemistry 2021

Experimental and DFT studies of gadolinium decorated graphene oxide materials for their redox properties and as corrosion inhibition barrier layer on Mg AZ13 alloy in a 3.5% NaCl environment

Palaniappan. N,[a*] Ivan Cole,[b*] A. Kuznetsov,[c] K.R. Justin Thomas,[d] Balasubramanian K.[e] Sivakumar Manickam[f]

[a]School of Chemical Sciences, Central University of Gujarat, India

[b]Advanced Manufacturing and Fabrication Research and Innovation, RMIT University, Melbourne, Victoria 3100, Australia

[c]Department of Chemistry, Universidad Técnica Federico Santa Maria, Campus Vitacura, Santiago, Chile

[d]Organic Materials Laboratory, Department of Chemistry, Indian Institute of Technology Roorkee, Roorkee 247667, India

[e]Department of Materials Engineering, Defence Institute of Advanced Technology (DU), Girinagar, Pune, India

[f]Petroleum and Chemical Engineering, Faculty of Engineering, Universiti Teknologi Brunei, Bandar Seri Begawan BE1410, Brunei Darussalam

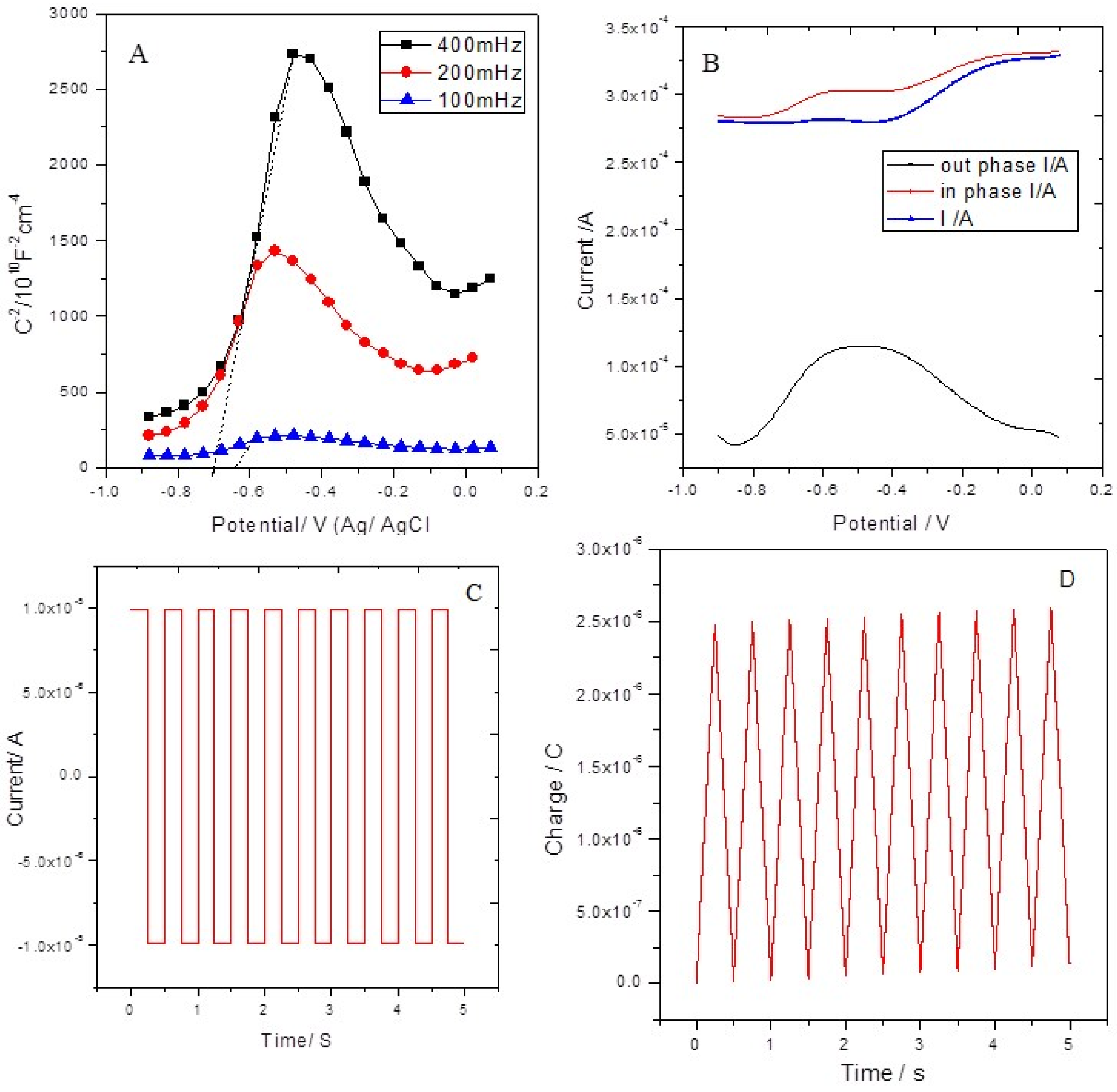

Figure S1. Motto-Schottky (a) AC-voltammetry (b) chronoamperometry (c) chronocoulometry (d) studies of Gd functionalized graphene oxide.

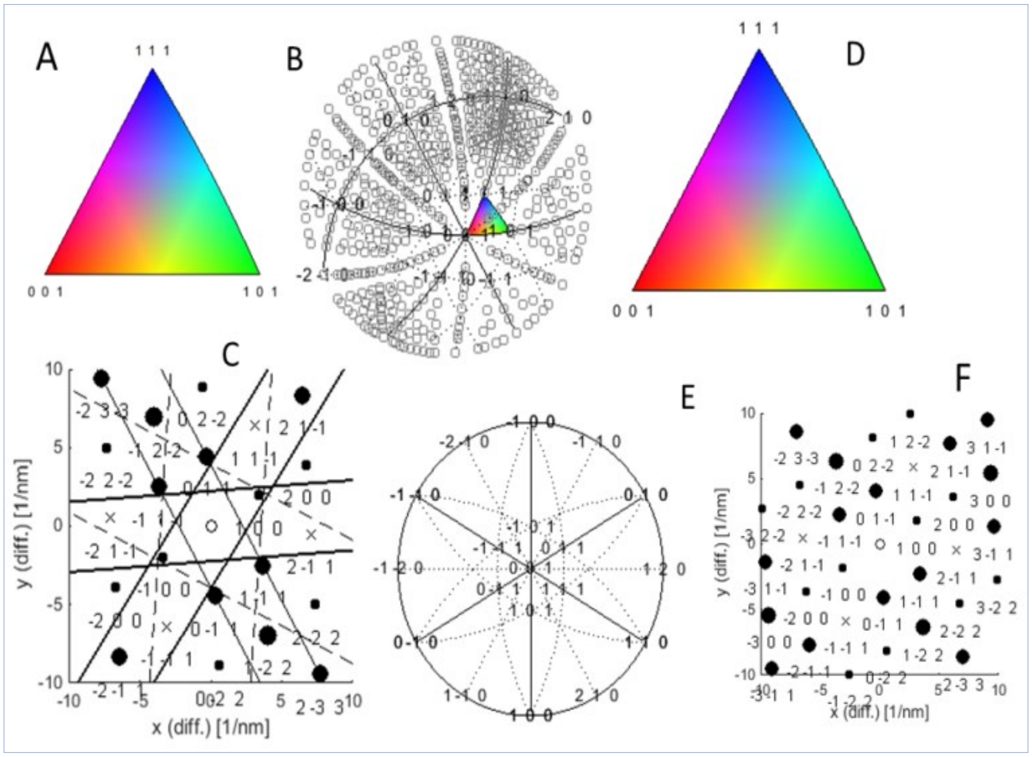

Figure S2. Epoxy and Gd+GO composite coated polycrystalline affected region of the Mg alloy surface.

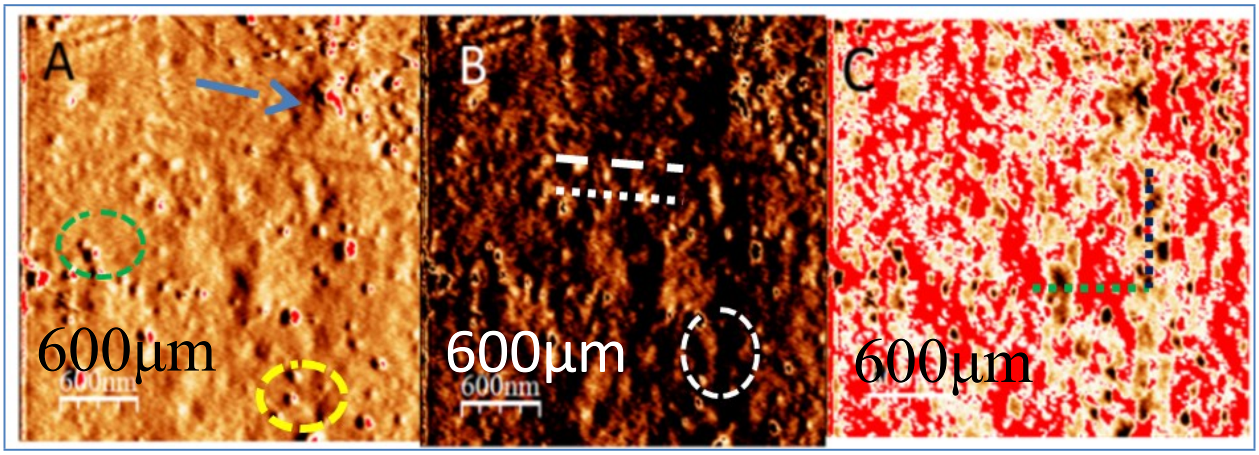

Figure S3. Topography of the epoxy coated Mg alloy immersed for 5 days in 3.5% NaCl solution.

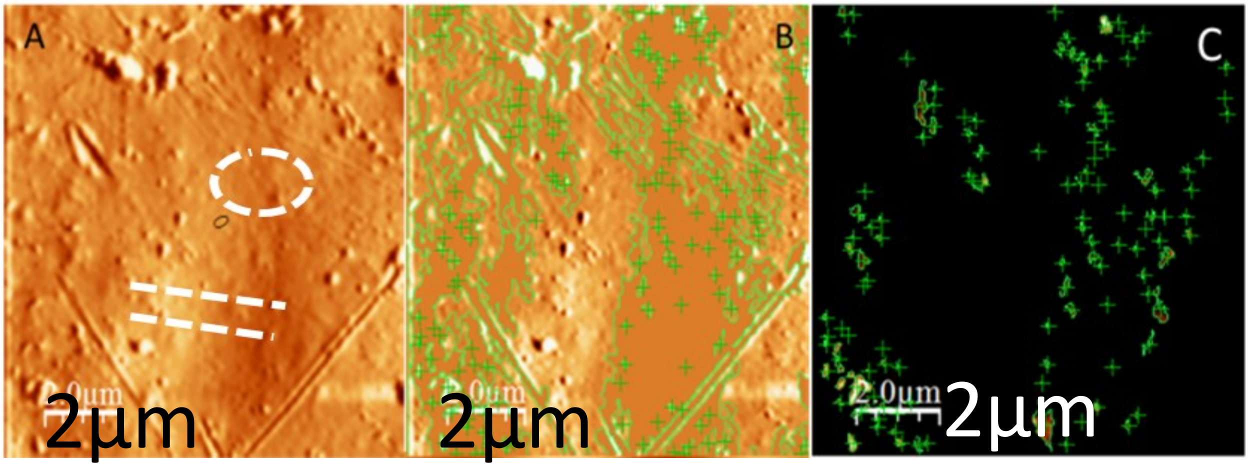

Figure S4. Topography of epoxy coated Mg alloy immersed for 5 days in 3.5% NaCl corrosion medium

S.no	$-E_{Corr}$ mV	$-I_{Corr}$mA	LP	η	RCtΩ	CdlµF
Epoxy	1.385	6.42	382		450	3.5710
Gd+GO	1.899	2.61	1408	59	700	0.1136

Table S1. Epoxy and Gd+GO coated Mg alloy corrosion inhibition efficiency

	HOMO	LUMO	ΔE	I	A	χ	[illegible]	σ	ω
					NEUTRAL				
A	-0.21757	-0.16128	0.05629	0.21757	0.16128	0.189425	0.02815	35.524	0.63733
B	-0.21716	-0.16033	0.05683	0.21716	0.16033	0.188745	0.02842	35.1865	0.62675
					PROTONATED				
A	-0.22866	-0.17520	0.05346	0.22866	0.17520	0.20193	0.02673	37.4111	0.76273
B	-0.22859	-0.17475	0.05384	0.22859	0.17475	0.20167	0.02692	37.1471	0.7554

Table S2. Global reactivity parameters computed for the Gd-GO system (with the implicit effects from water), for the neutral and protonated models (A.U.): HOMO-LUMO gap ΔE, ionization potential I, electron affinity A, global electrophilicity χ, global hardness η, global softness σ, and global nucleophilicity ω. A = α-HOMO & LUMO, B = α-HOMO & LUMO.
